# Myostatin and its Regulation: A Comprehensive Review of Myostatin Inhibiting Strategies

**DOI:** 10.3389/fphys.2022.876078

**Published:** 2022-06-23

**Authors:** Mohammad Hassan Baig, Khurshid Ahmad, Jun Sung Moon, So-Young Park, Jeong Ho Lim, Hee Jin Chun, Afsha Fatima Qadri, Ye Chan Hwang, Arif Tasleem Jan, Syed Sayeed Ahmad, Shahid Ali, Sibhghatulla Shaikh, Eun Ju Lee, Inho Choi

**Affiliations:** ^1^ Department of Family Medicine, Gangnam Severance Hospital, Yonsei University College of Medicine, Seoul, South Korea; ^2^ Department of Medical Biotechnology, Yeungnam University, Gyeongsan, South Korea; ^3^ Research Institute of Cell Culture, Yeungnam University, Gyeongsan, South Korea; ^4^ Department of Internal Medicine, College of Medicine, Yeungnam University, Daegu, South Korea; ^5^ Department of Physiology, College of Medicine, Yeungnam University, Daegu, South Korea; ^6^ School of Biosciences and Biotechnology, Baba Ghulam Shah Badshah University, Rajouri, India

**Keywords:** myostatin, skeletal muscle, MSTN inhibitors, natural compounds, peptides

## Abstract

Myostatin (MSTN) is a well-reported negative regulator of muscle growth and a member of the transforming growth factor (TGF) family. MSTN has important functions in skeletal muscle (SM), and its crucial involvement in several disorders has made it an important therapeutic target. Several strategies based on the use of natural compounds to inhibitory peptides are being used to inhibit the activity of MSTN. This review delivers an overview of the current state of knowledge about SM and myogenesis with particular emphasis on the structural characteristics and regulatory functions of MSTN during myogenesis and its involvements in various muscle related disorders. In addition, we review the diverse approaches used to inhibit the activity of MSTN, especially *in silico* approaches to the screening of natural compounds and the design of novel short peptides derived from proteins that typically interact with MSTN.

## Introduction

Skeletal muscle (SM) is the largest organ, comprising ∼40% of total body weight, and one of the most dynamic and plastic tissues in the human body (Holmberg and Durbeej, 2013). These highly dynamic plastic tissues constitute 50–75% of body protein content and perform a large number of crucial body functions such as movement, temperature control, and maintaining glucose levels (Frontera and Ochala, 2015). Muscle satellite cells (MSCs) are multipotent precursor cells that are found between the sarcolemma and the basal lamina and provide anatomical and functional stability, preserving SM integrity (Lee et al., 2018). MSCs are capable of self-renewing and developing differentiated progeny. In particular, the proliferation and differentiation of MSCs to myotubes via the myogenic program relies on the coordinated regulations of paired box transcription factors (Pax3/Pax7) and the basic helix-loop-helix (bHLP) family of transcription factors (myogenic factor 5, Myf5; myogenic differentiation, MyoD; and myogenin, MYOG). During the gradual muscle loss associated with muscular dystrophy (MD) or aging, MSC activity is commonly impaired due to imprecise asymmetrical division or abnormal transcriptional control (Bianchi et al., 2020).

Aging is characterized by a progressive loss of muscle mass, leading to a loss of muscular strength (Cartee et al., 2016). Apart from aging, the loss of muscle mass is also associated with several other disease conditions such as cancer, chronic obstructive pulmonary disease (COPD), muscular dystrophies, acquired immune deficiency syndrome (AIDS), immune disorders, congestive heart failure, etc. (Fanzani et al., 2012). Furthermore, sarcopenia, a condition that impairs physical ability and metabolism, is also linked to age-related loss of SM mass and function (Curtis et al., 2015).

Myostatin; also known as growth differentiation factor 8 (GDF8) has been well reported to negatively regulate muscle growth and size (Carnac et al., 2007; Chen et al., 2021). The putative involvement of MSTN in muscle atrophy has been documented in several studies, prompting interest in MSTN as a therapeutic target to counteract muscle loss in patients with a range of muscle-wasting conditions (Baczek et al., 2020; Sartori et al., 2021). MSTN-deficient mice were found to have 2 to 3 times the SM mass of wild-type mice, which indicated MSTN acts as a negative regulator of muscle cells *in vivo* (McPherron et al., 1997). MSTN inhibition is also regarded as a crucial therapeutic target in the context of enhancing muscle strength and insulin sensitivity (Camporez et al., 2016).

MSTN inhibition is considered to be a potentially effective means of addressing the issue of muscle loss. Computational methods are widely used to discover novel inhibitors in a quick and cost-effective manner, typically through peptide design and compound screening. Usually, peptides are generated based on the 3D structures of protein complexes (Baig et al., 2018). Peptide fragments are often created from the interacting residues of protein-protein interactions (PPIs), which are central considerations in rational drug design (Baig et al., 2016). Computational screening of large compound collections against the binding sites of target proteins often results in the rapid identification of potential ligands. Virtual screening (VS.) is usually conducted using structure- and ligand-based approaches (Baig et al., 2016).

Other TGF-β superfamily members, in addition to MSTN, are documented to be effective negative muscle regulators, notably “activin A” being the second negative muscle regulator. Latres et al. (2017) found that MSTN and “activin A” adversely affect muscle development and function, and that blocking both of these ligands with antibodies resulted in a large increase in muscle and lean body mass in mice and monkeys, with a better therapeutic window than inhibiting all TGF-ligands.

The role played by MSTN in the development and growth of SM, and the mechanism by which it regulates the myogenic process are discussed in this review together with its role in different diseases and the strategies used to inhibit its activity [from natural compounds to myostatin inhibitory proteins (MIPs)]. In addition, we also review the state-of-the-art *in silico* approaches used to design MSTN inhibitors based on the structures of its interacting proteins.

## Skeletal Muscle and Myogenesis

SM is composed of muscle fiber with a unique structure, which mainly consists of actin and myosin filaments that allow muscles to contract and relax. Each muscle fiber represents a muscle cell, which has a fundamental cellular unit known as the sarcomere. Fascicules are formed by bundles of myofibers, and muscle tissue is formed by bundles of fascicles, with each layer being contained by the ECM and maintained by cytoskeletal networks (Lieber and Friden, 2000). Thus, SM is responsible for body movement and posture. In addition, SM physically protects soft tissues, and internal organs, and maintains body temperature by producing heat using the energy generated during muscle contraction (Argilés et al., 2016).

The SMs present a strong capacity to regenerate, even after serious damage caused by heavy exercise, mechanical laceration, disease, or induced under experimental circumstances, e.g., by crushing or injecting cardiotoxin (CTX), begin a cascade of episodes leading to the muscle restoration (Collins and Morgan, 2003; Guardiola et al., 2017). MSCs divide symmetrically to increase their number, or asymmetrically to produce cohorts of committed satellite cells and consequently progenitors after they have been activated. Myogenic progenitors multiply and eventually differentiate by fusing with other myogenic progenitors or injured fibers in order to restore fiber integrity and function (Dumont et al., 2015; Dueweke et al., 2017).

## Regulation of Myogenesis

### Transcription Factors

MSCs present in SM originate from multipotent mesodermal cells. Like embryonic progenitors, the progression of MSCs along the myogenic lineage commences with the co-expressions of Pax3 and Pax7 and a family of bHLP transcription factors referred to as myogenic-regulatory factors (MRFs), such as Myf5, MyoD, Mrf4, and MYOG (Jan et al., 2016). These four MRFs are the fundamental constituents of the myogenic pathway. Myf5, the determining factor of myoblast, is expressed before commitment to myogenic fate. MyoD, which is induced by Myf5, drives cells to the myogenic lineage, and MYOG seems to work downstream of Myf5 and MyoD, and it is necessary for the establishment of the myogenic lineage as well as terminal differentiation of myoblasts (Lindon et al., 1998; Tapscott, 2005; Londhe and Davie, 2011; Ganassi et al., 2020).

When activated, MSCs undergo asymmetric division during muscle regeneration to give rise to two self-renewal daughter cells or emerge to form non-committed stem cells (Myf5-) for self-renewal or committed (Myf5^+^) cells (Kuang et al., 2008). The up-regulation of MyoD expression in activated MSCs (Pax7^+^/Myf5^+^) causes them to proliferate to generate myoblasts (Shefer et al., 2006). On the other hand, a decline in Pax7 expression in MyoD primed myoblasts marks their withdrawal from the cell cycle and entry into differentiation (Zammit et al., 2004). Collectively, the transcriptional network regulates the progression of the MSC lineage from origin to myogenic specification, differentiation, and fusion to produce myoblasts.

### Hormones

The regulation of muscle growth also involves different hormones. A balance between the secretions of growth hormone (GH) and testosterone was found to be a prerequisite for optimizing muscle growth (Jan et al., 2016). Testosterone controls the sizes and numbers of muscle fibers by stimulating the longitudinal growth of muscle fibers. Though testosterone is associated with enhanced MSC proliferation and differentiation, estrogen influences their trans-differentiation, whereby lipids accumulate in differentiating myotubes (Wheeler and Koohmaraie, 1994). As a result of stimulation of insulin-like growth factor-1 (IGF-1) production via the liver JAK-STAT pathway, GH stimulates muscle fiber hypertrophy by enhancing the synthesis of proteins associated with MSC proliferation (Velloso, 2008). IGF-1 is an important hormone for muscle development and strength and helps in the proliferative efficiency of MSCs. Reportedly, TGF-β1 acts as a multifunctional cytokine that helps regulate muscle repair by activating MSCs (Delaney et al., 2017). Oxytocin injection rapidly enhanced muscle regeneration in young animals by aged MSCs and their proliferation by activating the MAPK/ERK pathway (Elabd et al., 2014).

GH-mediated conversion of thyroid hormone (TH) thyroxine (T4) to triiodothyronine (T3) helps their distribution to different tissues via binding to thyroxine-binding globulin, albumin, or transthyretin (TTR) (Alshehri et al., 2015). TTR-based T4 distribution was found to promote myoblast differentiation by regulating the expressions of myosin light chain 2 (MYL2) and the calcium channel genes Cav1.1 and Cav3.1 (Lee et al., 2013). We recently reported that during myoblast differentiation, TTR maintains muscle homeostasis via the unique TH shuttle mechanism. Furthermore, we postulated a unique mechanism for T4 and T3 absorption and release in myoblasts, as well as the role of TTR as an intracellular T4 sensor during myogenesis. (Lee et al., 2019).

## Extracellular Matrix

The extracellular matrix (ECM) is a complex structure comprised of different structural molecules that provide mechanical support to cells and maintain biochemical signaling (Zhang et al., 2021). ECM proteins such as collagen IV and VI, laminins, and their receptors (e.g., integrin α7β1 and dystroglycan) have been reported to play crucial roles in SM development and to be responsible for SM homeostasis (Thorsteinsdóttir et al., 2011; Ahmad et al., 2020a; Ahmad et al., 2020b). ECM components interact with and regulate the MSC niche in muscle fibers, and alterations or inadequacies in the components of SM ECM can have dramatic effects on the characteristic functions of MSCs such as activation, self-renewal, proliferation, and differentiation (Thomas et al., 2015).

Some ECM proteins bind and modulate the function of MSTN, especially fibromodulin (FMOD), decorin, fibronectin, and laminins (Miura et al., 2010). Earlier, we investigated several ECM proteins, namely, FMOD (Lee et al., 2016; Lee et al., 2018), matrix gla protein (Ahmad et al., 2017), and dermatopontin (Kim et al., 2019), that play vital roles in the regulation of myogenesis. MSTN is known to inhibit the transcription factors Pax7, MYOD, and MYOG and thereby, regulate MSC proliferation and differentiation (Joulia-Ekaza and Cabello, 2006; McFarlane et al., 2008). Interestingly, it was observed FMOD bypassed the inhibitory effects of MSTN and maintained its transcriptional activity. We showed that FMOD directly bound with MSTN in myoblast differentiation by co-immunoprecipitation. Furthermore, PPIs between FMOD and MSTN and its receptor (Activin receptor type-IIB, ACVRIIB) showed that FMOD effectively reduced the interaction between MSTN and ACVRIIB (Lee et al., 2016).

Intracellular aggregation of methylglyoxal, a precursor of advanced glycation end-products (AGEs), and subsequent glycation of biomolecules impaired ECM remodeling, and curcumin and gingerol have been reported to reduce the impact of AGE on myoblasts (Baig et al., 2017). Moreover, enhanced AGE production and consequent RAGE (AGE receptor)-AGE interaction hinders the muscle development program. We also found by *in silico* analysis that the MSTN-ACVRIIB interaction is reduced by curcumin or gingerol. Protein-ligand (curcumin/gingerol and MSTN) and protein-protein interactions (MSTN and ACVRIIB) studies were carried out to explore the effect of curcumin and gingerol in the myogenesis processes. MSTN was found to have interacted with ACVRIIB with an energy score of −56.99. However, the free energy of MSTN to ACVRIIB binding fell to −46.55 and −47.26, correspondingly, for MSTN-curcumin and MSTN-gingerol complexes, showing that curcumin and gingerol interfere with MSTN-ACVRIIB interaction (Baig et al., 2017).

## Myokines

SM produces several bioactive proteins, including cytokines, and numerous other peptides collectively called “myokines”. Skeletal myofibers produce a plethora of myokines, which exert auto-, para, and/or endocrine effects. Since myokine secretion is generally regulated by exercise, it has various advantageous effects on metabolic, cardiovascular, and mental health (Manole et al., 2018). Myokines are known to be involved in MSC activation and regulate their major functions, for example, they augment proliferation and differentiation rates (Mandai et al., 2017). The roles of myokines in the SM milieu appear to be directed toward MSC regulation at different stages, for instance, MSTN and growth differentiation factor 11 (GDF11) down-regulate MSC activation, proliferation, fusion, and differentiation, while IGF-1 promotes MSC fusion and differentiation (Mandai et al., 2017; Suh and Lee, 2020b).

Interleukin (IL)-6 plays a pleiotropic function in multiple tissues and organs. SM produces and secretes IL-6 during prolonged exercise, and is thus reflected as myokines (Munoz-Canoves et al., 2013). Local IL-6 production increase MSCs activation and promote the regeneration of myotube (Munoz-Canoves et al., 2013). Besides, IL-6 treatment has been found to enhance MSCs proliferation by controlling the cyclin D1 and c-myc genes (Serrano et al., 2008). The importance of IL-6 in myogenic differentiation has been confirmed as myoblast obtained from IL-6 null mice exhibits reduced fusion ability *in vitro* (Hoene et al., 2013). Like IL-6, leukemia inhibitory factor (LIF) has also been identified as a myokine, released by SM in response to exercise (Broholm and Pedersen, 2010; Pedersen and Febbraio, 2012). LIF regulates MSCs proliferation both in mice and humans. Exogenous LIF promotes the proliferation of human myoblast by inducing the transcription factors JunB and c-Myc (Broholm et al., 2011). In addition, LIF has also been found to induce myoblast differentiation (Yang et al., 2009).

Interleukin-15 (IL-15) is highly expressed in SM and has anabolic effects on SM protein dynamics (Quinn et al., 2002). IL-15 mRNA expression is up-regulated during myoblast differentiation and its administration inhibits the white adipose tissue deposition in rodents (Quinn et al., 2005). Also, IL-15 treatment decreased muscle protein degradation and SM wasting in an *in vivo* rat model of cancer cachexia (Carbo et al., 2000). Furthermore, reduced exercise endurance has been reported in IL-15−/− mice, however, enhanced exercise induction has been found in SM-specific IL-15-transgenic mice (Quinn et al., 2013; Quinn et al., 2014). Altogether, the above studies indicate that IL-6, IL-15, and LIF appeared as a vital myogenesis controllers, functioning during both myoblast proliferation and differentiation.

## Myostatin

The MSTN protein sequence includes a secretion signal sequence, a proteolytic processing site, and a carboxy-terminal region with a conserved pattern of nine cysteine residues, all of which are shared by TGF-superfamily members. MSTN activation requires proteolytic cleavages of the precursor protein by a furin family enzyme and BMP1/Tolloid matrix metalloproteinase (Huang et al., 2011). Natural MSTN mutations in increased SM mass in many species including humans and similar results have been observed in MSTN null experimental mice (Amthor et al., 2007). During embryogenesis, MSTN is produced by cells in the myotome and developing SM and regulates the overall amount of muscle fibers formed. In adults, MSTN is secreted by SM, circulates in the blood, and inhibits muscle fiber growth (Lee, 2012).

### Structure of MSTN

MSTN is translated as a precursor protein, which undergoes several proteolytic processing events that result in the formation of active, mature MSTN (Qian et al., 2015). Initially, the amino(N)-terminal signal sequence is removed by a signal peptidase to form Pro-MSTN, and dimerization follows due to disulfide bond formation near carboxy(C)-termini. Subsequently, furin cleaves the dimer at its proteolytic processing site RXXR site. C-terminus cleavage results in an N-terminal propeptide with an N-linked glycosyl group and a receptor-binding domain at the C-terminal. Latent MSTN complex forms when the N-terminal propeptide binds the C-terminal region noncovalently through a crucial peptide sequence, which prevents MSTN from binding to its receptor. In the last stage, BMP-1/TLD cleaves the propeptide, which leads to the release of mature MSTN. Increased muscle growth in adult mice was attributed to an inability to cleave the latent complex (Wolfman et al., 2003).

Like other members of the pro-TGF-β superfamily, pro-MSTN is a homodimer comprised of two identical disulfide-linked subunits. Each chain consists of 109 amino acid residues containing a pro-domain (N-terminal) and a smaller growth factor (GF) domain (C-terminal). As found in other members of the TGF-β superfamily, the GF domain of MSTN contains a cystine-knot motif and four antiparallel β-strands referred to as “fingers”. The two identical GF domains of MSTN are connected by their concave “palms”, which are covalently linked to each other by disulfide bonds between C339 residues in the wrist region. The pro-domain contains N-terminal “forearm” helices, which grasp mature GF, and a globular “arm/shoulder” domain, which sits on top of the mature GF protomers (Cotton et al., 2018). Each MSTN monomer has four intermolecular disulfide bonds, three of which are involved in cysteine knot formation. When the two monomers of MSTN come together in an antiparallel direction they generate convex or concave surfaces. The cysteine knots and dimerization are the major determinants of MSTN stability. Due to its similarity with activin class members (∼40%) and its binding to activin receptors and inhibitors (follistatin; FST), MSTN has long been considered to be a member of the activin class, as it has been shown to interact with ACVRIIB and ACVRIIA, as well as FST (Lee and McPherron, 2001). However, later x-ray structural analysis demonstrated that it is a member of the TGF-β superfamily, though it exhibits remarkable differences in the N-terminal region and in the region preceding the wrist helix (Cotton et al., 2018).

## Signaling Pathways

**FIGURE 1:**
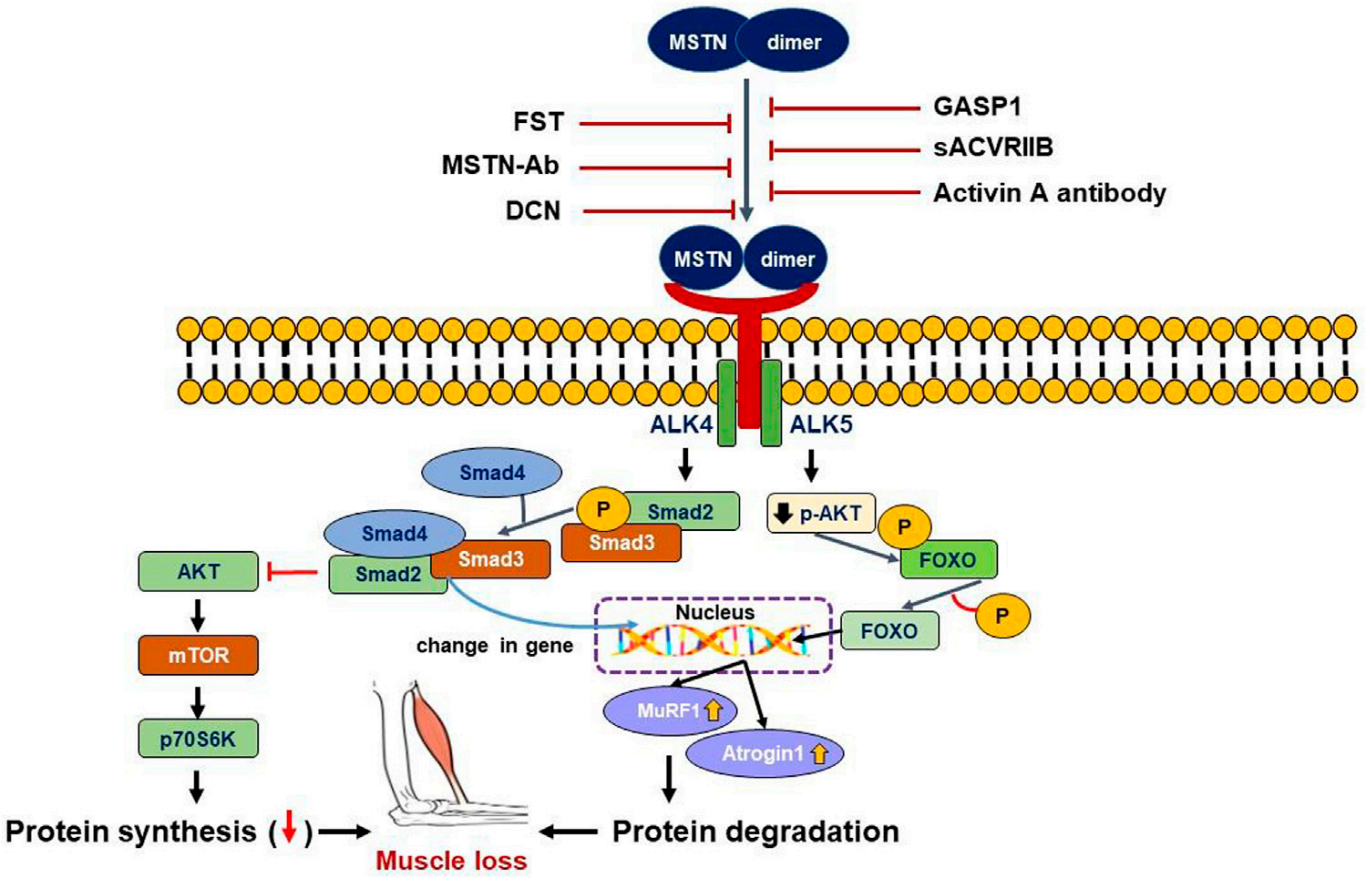
Smad and non-Smad mediated signaling pathway of MSTN. MSTN binds to the ACVRIIB and ALK4/5 complex resulting in successive phosphorylation of Smad2/3, leading to its binding with Smad4 and translocation of the complex to the nucleus. Non-Smad signaling, on the other hand, tends to suppress the AKT intracellular signaling pathways. Both Smad and non-Smad mediated signaling cause gene transcriptional alterations in the nucleus, as well as activation of muscle atrophy marker genes (MuRF1 and Atrogin1), resulting in muscle loss. Extracellularly, MSTN pathway inhibitors can bind MSTN directly or bind its receptor complex to prevent MSTN from interacting with its receptor complex and triggering downstream signals.

The mechanisms of MSTN-induced SM loss are mediated by reduced protein synthesis and/or enhanced protein catabolism (Elliott et al., 2012). MSTN decreases protein synthesis by inhibiting the Akt/mTOR signaling pathway and induces muscle atrophy by promoting the transcriptions of atrophy-related genes (atrogenes). MSTN signaling pathways can be divided into Smad and non-Smad mediated pathways **(** Figure 1).

### The Smad-Mediated Pathways

Mature MSTN contains a disulfide-linked dimer of the C-terminal domain and is 100% identical in humans, mice, rats, pigs, chickens, and dogs. The C-terminal dimer of mature MSTN binds to ACVRIIB and results in the recruitment and activation of activin type I receptors (Alk4 or Alk5), which in turn promote the activations of Smad 2 and Smad 3 to inhibit myoblast differentiation. Activation of MSTN signaling inhibits Akt phosphorylation by IGF-1 and the IGF-1-induced protein synthesis pathway possibly under Smad 3 mediation (Morissette et al., 2009). Inhibition of the MSTN-ACVRIIB-ALK4/ALK5-Smad2/3 pathway is strongly associated with muscle hypertrophy in adults, and Smad3 also increases E3 ligase atrogin-1 expression, which increases protein catabolism by the proteasome ubiquitination pathway (Goodman et al., 2013). Although Smad2/3 plays a predominant role in the protein catabolic pathway induced by MSTN, Smad3 null mice also exhibit MSTN-induced SM atrophy. Furthermore, MSTN overexpression was found to repress the differentiation of myoblasts by inhibiting MyoD and MyoD expression through MEK/Erk1/2 pathways (myogenic differentiation suppression pathways) (Yang et al., 2006). Thus, MSTN regulates muscle mass by acting through various signaling pathways that regulate muscle growth.

### The Non-Smad Pathways

MSTN activates the JNK/Erk 1/2 (c-Jun N-terminal kinase/Erk 1/2) signaling pathway in proliferating and differentiating C2C12 cells (Huang et al., 2007). Philip et al. reported MSTN activates p38 MAPK through the TAK1-MKK6 cascade independently of Smad activation in proliferating A204 and C2C12 cells and that p38 MAPK plays an important role in the MSTN-regulated inhibition of myoblast proliferation (Philip et al., 2005). In another study, MSTN was found to act upstream of Wnt pathway components and suppress Wnt4 expression, which is capable of stimulating MSC proliferation. Therefore, inhibition of Wnt signaling downregulated MSC proliferation (Steelman et al., 2006). Altogether, these findings suggest the complexity of MSTN signal transduction is probably due to the involvement of different signaling pathways and that the precise integration of these pathways underlies the growth inhibitory effects of MSTN.

## Pathologic Roles of MSTN

Muscle wasting is associated with cancer-related cachexia, age-related sarcopenia, and metabolic diseases such as obesity and diabetes, which are all directly related to morbidity and mortality. Associations between MSTN and muscle-wasting conditions have been investigated at the clinical level (Consitt and Clark, 2018). Plasma MSTN has been reported to increase with age, and its association with the prevalence of sarcopenia was found to be stronger in women (Bergen et al., 2015). In particular, plasma MSTN has been suggested to be a possible marker for the early diagnosis of cachexia in women with medullary thyroid carcinoma (Hedayati et al., 2016). Furthermore, the expression of MSTN in SM was significantly greater in gastric cancer patients before cachexia became clinically apparent, but this was not observed in lung cancer patients (Aversa et al., 2012). In another study, MSTN expression in the SMs of patients with gastric cancer was no different from that observed in healthy controls and was not associated with weight loss (D'Orlando et al., 2014). Increased protein degradation is probably a primary cause of pathologic muscle depletion, and it has been reported that protein degradation was enhanced in an experimental model of cancer cachexia and associated with muscle atrophy. In preclinical investigations of cancer cachexia, excessive proteolysis, particularly via autophagy and the ubiquitin-proteasome system, and low protein synthesis in SM have been documented (Sandri, 2016).

### Diabetes and Obesity

Studies have explored the impact of MSTN on insulin resistance, and several studies conducted using mouse models have provided evidence that the absence of MSTN has significant effects on metabolism, that is, it improves insulin sensitivity and reduces obesity (Amor et al., 2019). Many studies have suggested that MSTN has a substantial impact on metabolism and may contribute to the development of obesity and diabetes (Allen et al., 2008; Allen et al., 2011). MSTN protein secretion was higher from the cultured myotubes of obese insulin-resistant subjects. High MSTN expression was first reported in the SMs of morbidly obese patients, whereas low expression was associated with subsequent fat loss (Hittel et al., 2009). In addition, MSTN expression and insulin sensitivity were found to be inversely proportional, and it was suggested a causal relationship exists between MSTN expression and insulin sensitivity (Hittel et al., 2010). In high-fat diet (HFD) mice, mutation-induced reductions in MSTN activity protected against obesity-induced insulin resistance (Wilkes et al., 2009). MSTN decreased insulin-induced GLUT4 membrane translocation and glucose absorption by inhibiting GLUT4 expression (Ahmad et al., 2018; Liu et al., 2018).

### Cancer Cachexia and Sarcopenia

Because SM is largely composed of proteins, an imbalance between protein synthesis and degradation sensitively affects muscle mass, and reductions in muscle mass may lead to functional disability and an increase in the risk of injury and mortality. Cachexia and sarcopenia are two representative conditions that are closely related to gradual muscle loss and its inevitable consequences. Although the mechanisms of muscle loss are not clearly defined in either condition, the results obtained from experimental rodent models and clinical trials indicate that MSTN inhibition offers a promising means of controlling muscle loss and cancer-related cachexia (Sakuma and Yamaguchi, 2012).

Cachexia is a multifactorial syndrome associated with a chronic illness that causes involuntary weight loss due to reduced SM mass with or without fat mass loss. Cachexia is associated with chronic inflammatory disorders such as COPD, heart failure, chronic kidney disease, AIDS, sepsis, and most commonly cancer. The overall prevalence of cachexia due to any disease is around 1% among the patient population (i.e. approximately 9 million) (von Haehling and Anker, 2014). The most prominent clinical feature of cancer-related cachexia is SM loss due to anorexia and increased protein catabolism. Nutritional support does not reverse weight loss and the condition is treated using appetite-enhancing and/or anti-inflammatory drugs (Sadeghi et al., 2018).

Inflammation probably contributes to muscle atrophy by regulating the NF-κB signaling pathway, and conversely, the suppression of inflammation reverses muscle atrophy (Yu et al., 2017). In a study conducted on animals with tumor-induced cachexia, the expression of MSTN was up-regulated (Samant et al., 2017), and another study showed that blockage/inhibition of MSTN in animals with cancer cachexia prevented muscular atrophy without affecting tumor growth (Aversa et al., 2017). Thus, it is believed that increased MSTN expression is responsible for the progression of cancer and cancer-associated cachexia. Furthermore, it was observed that genetic deletion of MSTN or inhibition of its expression using anti-sense oligonucleotide preserved muscle mass (Gallot et al., 2014). The addition of a soluble antagonist of ACVRIIB that antagonizes MSTN signaling also reduced cancer-associated cachexia and reversed muscle wasting (Zhou et al., 2010). In a recent review, Hulmi et al. reviewed the numerous anti-cachectic benefits of ACVR2 inhibition in preclinical cancer models and in combination with anticancer therapies (Hulmi et al., 2021). The administration of this antagonist to mice with Lewis lung carcinoma or transfected with colon-26 cells improved muscle strength and reversed muscle wasting and prolonged survival in mice with a C26 tumor (Zhou et al., 2010; Busquets et al., 2012; Hatakeyama et al., 2016). MSTN levels and MSTN-mediated signaling pathways were found to be upregulated in experimental cancer cachexia and cancer patients even before the development of cancer cachexia (Costelli et al., 2008). However, plasma levels of MSTN are not always correlated with muscle loss in human cancer patients (Loumaye et al., 2015), and genetic deletion of MSTN or its acute inhibition using trichostatin A or FST does not always have a preventive effect against cancer cachexia in experimental rodents (Bonetto et al., 2009; Benny Klimek et al., 2010). Antibody against MSTN failed to elicit any significant clinical benefit in muscle dystrophy or pancreatic cancer patients (Wagner et al., 2008; Golan et al., 2018).

Sarcopenia is the result of a decline in the number of motor units and muscle fiber atrophy and is more prevalent these days due to the increasing number of elderly (Stoever et al., 2017). Although it is well known that reduced protein synthesis and/or increased protein degradation induces SM atrophy, reports regarding the underlying molecular pathways are inconsistent. Currently, no approved therapy is available for treating sarcopenia. Nevertheless, numerous reports have demonstrated that MSTN is a potential therapeutic target (White and LeBrasseur, 2014).

Moreover, reported relations between MSTN and muscle mass and aging also vary. In healthy older men, lower serum MSTN levels were linked to lower SM mass, but not in women (Peng et al., 2018). It is also evident that serum MSTN does not differ in young and sarcopenic elderly men (Ratkevicius et al., 2011). On the other hand, serum MSTN levels are elevated in elderly people and inversely correlated with lean mass. This discordance may stem from the technical limitations of enzyme-linked immunosorbent assays or radioimmunoassays used to discriminate between active and inactive MSTN or between MSTN and the similar protein (GDF-11) (Yarasheski et al., 2002). In SM, although MSTN mRNA levels are not correlated with age, MSTN protein levels are elevated in elderly subjects. Furthermore, MSTN protein levels are higher in the muscle tissues of elderly men than in healthy young men after acute muscle exercise (McKay et al., 2012).

Drug development targeting MSTN or its signaling pathways is being actively pursued. The amount of nuclear FOXO1 was increased in myotubes after MSTN treatment (McFarlane et al., 2006). FOXO1 and Smad2 were found to synergistically increase the MSTN mRNA expression and its promoter activity in the myotube of the C2C12 cell (Allen and Unterman, 2007). MSTN induced cachexia has been reported to be triggered by the activation of the ubiquitin-proteasome system (UPS) via FOXO1-dependent signaling, and it has been shown that increased MSTN expression is involved in the production of COPD-related muscle atrophy (Testelmans et al., 2010).

Ghrelin is largely produced in gastric oxyntic mucosa (DeBoer, 2011), and ghrelin treatment reduces proinflammatory cytokine release in cachexia patients (Kishimoto et al., 2012). Furthermore, increases in anabolic activity by ghrelin enhance GH release and reduce the effects of inflammation, which offers promise for the treatment of cachexia (Yanagi et al., 2018). In addition, ghrelin has been shown to prevent muscle atrophy in rats by enhancing AKT phosphorylation, suppressing the MSTN pathway, and activating myogenin and MyoD (Chen et al., 2015). Ghrelin formulations for parenteral administration are being developed (Garin et al., 2013). However, administration by injection over extended periods can result in poor patient compliance and therapy failure, and ghrelin is prone to enzymatic breakdown in blood when delivered intravenously (Brimijoin et al., 2016). Liposomes are frequently utilized as drug carriers due to their ability to encapsulate hydrophilic, amphiphilic, and lipophilic compounds (Pashirova et al., 2020), and the protective phospholipid layers of these systems protect ghrelin from pH, free radical, and enzymatic degradation *in vivo* (Sessa and Weissmann, 1968) and metabolism in the mucosal layer of the nasal cavity (Vieira and Gamarra, 2016). Ghrelin liposomes coated with chitosan are being developed for nose-to-brain administration for the treatment of cachexia (Salade et al., 2017). Furthermore, it has been shown that anionic liposomes can protect ghrelin from enzymatic breakdown by trypsin and carboxylesterase. Ghrelin interacts with lipid bilayers electrostatically and hydrophobically. Coating ghrelin with N-(2-hydroxy) propyl-3-trimethyl ammonium chitosan chloride enhanced mucin adsorption capacity (22.9%), with improved permeability via Calu3 epithelial monolayers recovering 10.8% of ghrelin in the basal compartment versus nonloaded ghrelin was used. Ghrelin can also be protected from metabolic enzymes in nasal tissues. Anionic liposomes coated with chitosan in dry powder form exhibited better mucin adhesion, ghrelin loadings, and enzymatic protection against trypsin, and reduced ghrelin degradation during storage at room temperature (Howick et al., 2018).

## MSTN Inhibition

Ever since its discovery, intensive research has been conducted to suppress the activity of MSTN using soluble activin type IIB (sACVRIIB) receptors, peptides or propeptides, small molecules, neutralizing antibodies, or MIPs (Table 1).

**TABLE 1:** List of MSTN inhibitors.

Category/type	Name	Stage	Function	References
Myostatin/ACVRIIB Antibodies	LY-2495655	phase 2 trial	increases lean mass in elderly people	Becker et al. (2015)
	MYO-029	phase 1/2 trial	used to treat DMD by binding to myostatin and inhibiting its function	Wagner et al. (2008)
	PF-06252616	phase 2 trial	induce muscle anabolic activity in the mdx mouse model of DMD	St Andre et al. (2017)
	ATA 842		increased muscle mass and muscle strength in young and old mice	Camporez et al. (2016)
	ACE-031	Phase 2 trial (terminated)	potential therapy for myopathies	Campbell et al. (2017)
	ACE-2494	Phase 1 trial	significant gain in muscle mass in Col1a1Jrt/+mice	Tauer and Rauch, (2019)
	ACE-083	Phase 2 trial	improve muscle mass in a variety of neuromuscular conditions	Glasser et al. (2018)
	Bimagrumab	Phase 2 trial	enhances differentiation of primary human skeletal myoblasts and increases SM mass in mice	Dutt et al. (2015)
Natural compounds	Epicatechin	phase 1/2a trial	enhances exercise capacity in mice	Gutierrez-Salmean et al. (2014)
	Sulforaphane	Phase 2 trial	repairs vascular smooth muscle cell dysfunction in age-related cardiovascular diseases and protects against skin aging	Bose et al. (2020)

### Antibodies

The injection of MSTN neutralizing antibodies is known to significantly increase muscle mass (by up to 17%) in aged mice. The administration of neutralizing antibodies targeting MSTN, such as LY-2495655, MYO-029, PF-06252616, ATA 842, and REGN1033/SAR391786, improves body metabolism as well as the increase of SM mass (Camporez et al., 2016; St Andre et al., 2017). Furthermore, these antibodies help increase muscle mass and strength while attenuating muscle atrophy and act by blocking the ability of MSTN to prevent the signaling of TGF-β family members (Latres et al., 2015).

### MSTN Receptor Proteins

ACVRIIB is a widely reported signaling receptor for several members of the TGF-β superfamily. ACVRIIB is involved in the negative regulation of muscle mass and is extensively distributed in SM, adipose tissues, and other organs. ACE-031 is a soluble form of ACVRIIB, and various studies on an Amyotrophic Lateral Sclerosis mouse model have shown a single dose of ACE-031 increases muscle mass and strength. This fusion protein of ACVRIIB and IgG1-Fc acts by binding to MSTN, and thus, disrupts its inhibitory effect (Campbell et al., 2017). Experiments on ACE-031 were subsequently suspended due to possible safety issues of epistaxis and telangiectasia.

### Peptides/Propeptides

The latent MSTN complex circulates in the blood and is subsequently converted into its mature form after the removal of associated propeptides (Lee and McPherron, 2001). The binding of MSTN by these propeptides prevents MSTN functioning, and this is considered potential means of increasing muscle mass (Yang et al., 2001). Soon after the removal of these propeptides by proteolytic cleavage and conversion of latent MSTN to its mature form, MSTN binds to its receptor (usually ACVRIIB) and initiates the signaling process that regulates muscle growth. At this stage, several MIPs inhibit the binding of MSTN to ACVRIIB.

### Myostatin Inhibitory Proteins

#### Follistatin

Follistatin (FST) is an extracellular cysteine-rich glycoprotein, which is structurally dissimilar to TGF-β family members, and it has been established that FST interrupts the activity of MSTN by binding to it and preventing MSTN binding to its receptor (Lee and McPherron, 2001; Liu et al., 2021). *In vivo* studies have reported that the overexpression of this glycoprotein has hypertrophic effects on mouse muscles similar to those observed in MSTN null mice (Winbanks et al., 2012). Moreover, a homozygous mutation in the FST gene reduces muscle mass, which suggests it plays an important role in the regulation of myogenesis (Lee et al., 2010; Liu et al., 2021).

#### FST-Related Gene

FST-related gene (FLRG) also known as FSTL3 protein, exhibits high homology to a 10-cysteine repeat of FST. *In vitro* studies have shown that like FST, FLRG binds to activin and BMPs to inhibit their biological activities (Tsuchida et al., 2001). Furthermore, endogenous latent MSTN complex largely circulates in association with propeptide and FLRG, which both act independently as negative regulators of MSTN, probably by preventing MSTN binding to its receptor (Thies et al., 2001). Reports suggest that FLRG potently inhibits MSTN activity in a concentration-dependent manner (Hill et al., 2002). Monovalent FSTL3-Fc fusion protein (mono-FSTL3-Fc) generated with knobs-into-holes technology has recently been reported to overcome the limitations of existing anti-myostatin therapies, as systemic administration of mono-FSTL3-Fc in mice resulted in muscle fiber hypertrophy and improved muscle mass *in vivo* (Ozawa et al., 2021a; Ozawa et al., 2021b).

##### Growth and Differentiation Factor-Associated Serum Protein

Growth and differentiation factor-associated serum protein-1 and -2 (GASP-1 and GASP-2) also importantly regulate the biological activity of MSTN (Lee and Lee, 2013). These two proteins are mostly expressed in adult tissues and have been well reported to induce small but significant increases in muscle mass in mice (Monestier et al., 2012). One of the multiple domains of GASP-1 is homologous to the 10-cysteine repeat of FST, whereas, in GASP-2, the FSD domain is responsible for MSTN binding. Recombinant GASP-1 binds directly to mature MSTN and its propeptide, and the inhibitory potential of GASP-1 is reduced when its C-terminal domains are removed (Hill et al., 2003).

##### Decorin and FMOD

Decorin (DCN) is a component of the MSTN signaling pathway and has been reported to antagonize the effects of MSTN. This member of the small leucine-rich proteoglycan gene family has been found to suppress MSTN activity efficiently and to enhance the differentiation and proliferation rates of myogenic cells (Kishioka et al., 2008). Reportedly, MSTN and decorin are produced at the same time in muscle cells (Nishimura et al., 2002). FMOD is known to be actively involved in the assembly of ECM and was recently reported to be a novel regulator of MSTN during myoblast differentiation by regulating the transcriptional activities of MSTN and other myogenic marker genes, which include myogenin (MYOG) and myosin light chain 2 (MYL2) (Lee et al., 2016). FMOD suppresses muscle aging by negatively regulating the MSTN gene or reducing the action of MSTN protein, while MSTN promotes muscle aging by positively regulating the expressions of the Atrogin1, CD36, and PPAR genes in muscle tissues (Lee et al., 2021a).

## Natural Compounds

### Epicatechin

(-)-Epicatechin (EC) is a flavonol, anti-oxidant, and bioactive stereoisomer of catechin that is used as a food supplement and found in cocoa and green tea (Gadkari and Balaraman, 2015). EC treatment also reduced MSTN expression and significantly increased the levels of myogenic marker genes responsible for muscle growth in the quadriceps muscles of mice, and it has been well-established aging is associated with higher MSTN levels but reduced levels of several myogenic genes such as FST, MYOG, and MyoD (Mafi et al., 2019). These findings suggest EC promotes SM development by inhibiting MSTN.

### Fructus Schisandrae

*Fructus Schisandrae* (FS; Schisandra chinensis) is a well-known traditional herb in Korea, China, and Japan. The dried fruit of this herb (called Baill) is used to enhance physical capacity and for its anti-inflammatory and anti-stress effects (Panossian and Wikman, 2008). It has been reported that FS extract helps increase SM mass. When administered to a mouse MD model that exhibited high MSTN mRNA levels, FS extract reduced these levels in a dose-dependent manner (Kim et al., 2015).

### Sulforaphane

Sulforaphane (SFN), which is found in cruciferous vegetables, is a bioactive isothiocyanate that inhibits the activity of histone deacetylases (HDACs) (Myzak et al., 2006). SFN significantly reduces MSTN expression in porcine MSCs and can inhibit HDAC activities and DNA methyltransferase expression (Fan et al., 2012).

### Astragalus Polysaccharide

*Astragalus polysaccharide* (APS; also known as Huang Qi) is a well-known extract of *Astragalus membranaceus* (Fisch.) Bge (AMB) (Fu et al., 2013). Injection of APS into the SMs of non-insulin-dependent type 2 diabetic KKAy mice ameliorated insulin resistance and hyperglycemia, and reduced MSTN levels in SM, which demonstrated APS might improve insulin sensitivity and reduce SM MSTN levels by downregulating the ROS-ERK-NF-κB pathway (Liu et al., 2013).

### Glycyrrhiza Uralensis

*G. uralensis* is native to Asia and is used as a medicinal herb, sweetener, and in traditional Chinese medicine (Ji et al., 2016). Recently, we reported that *G. uralensis* inhibits MSTN expression and promotes myogenesis. In addition, liquiritigenin, tetrahydroxymethoxychalcone, and licochalcone B isolated from the EtOAc fraction of *G. uralensis* enhanced myoblast proliferation and differentiation, and liquiritigenin enhanced muscle regeneration in injured muscles (Lee et al., 2021b). These findings show that *G. uralensis*-derived compounds have therapeutic potential for the management of muscle-related disorders.

## Developmental Approaches Used to Design MSTN Inhibitors

**FIGURE 2:**
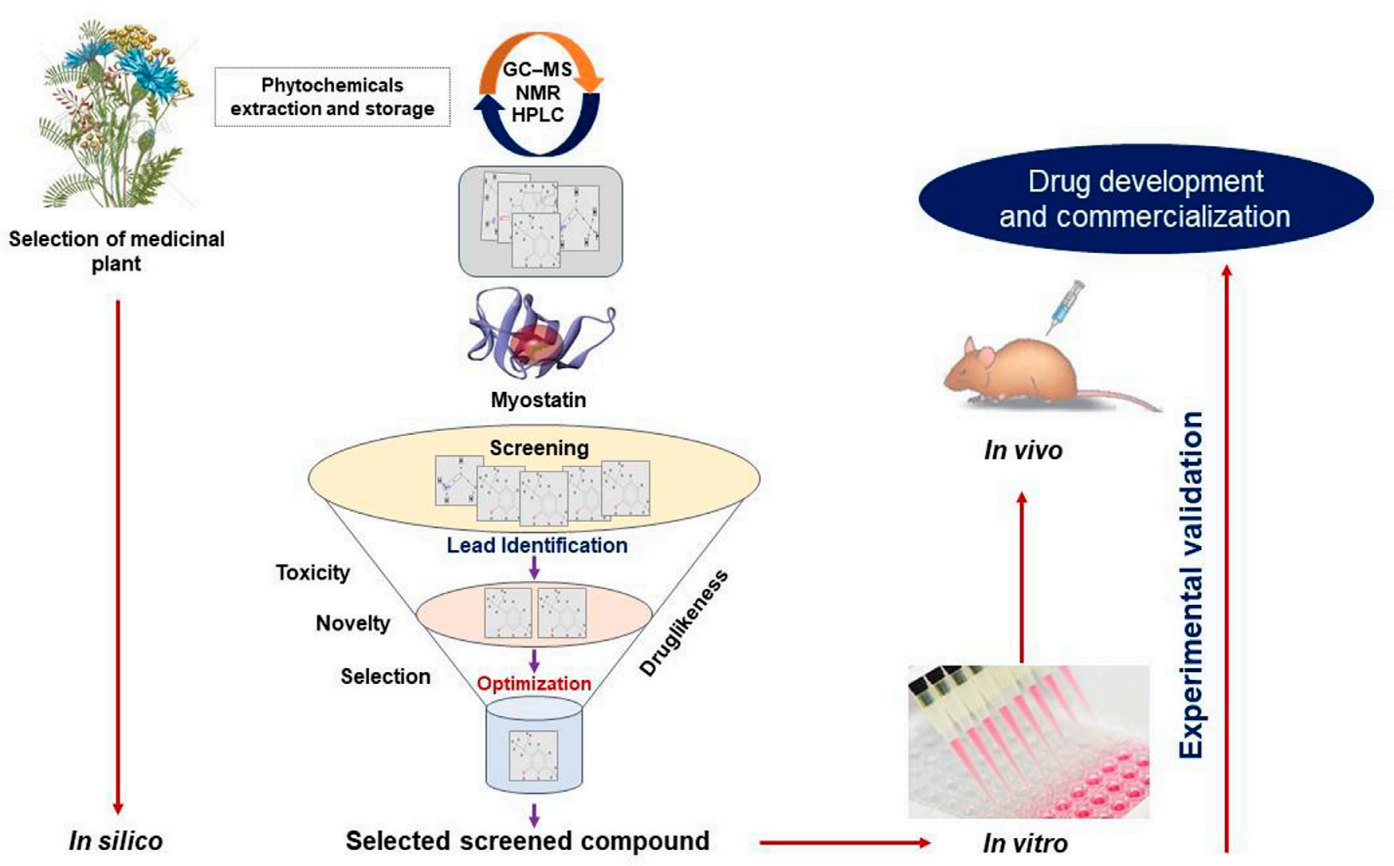
A typical approach for the conventional drug design and development strategy. An overview of the techniques for identifying MSTN inhibitors using in silico (virtual screening, molecular docking, ADMET, and so on), *in vitro*, and *in vivo* approaches.

**FIGURE 3:**
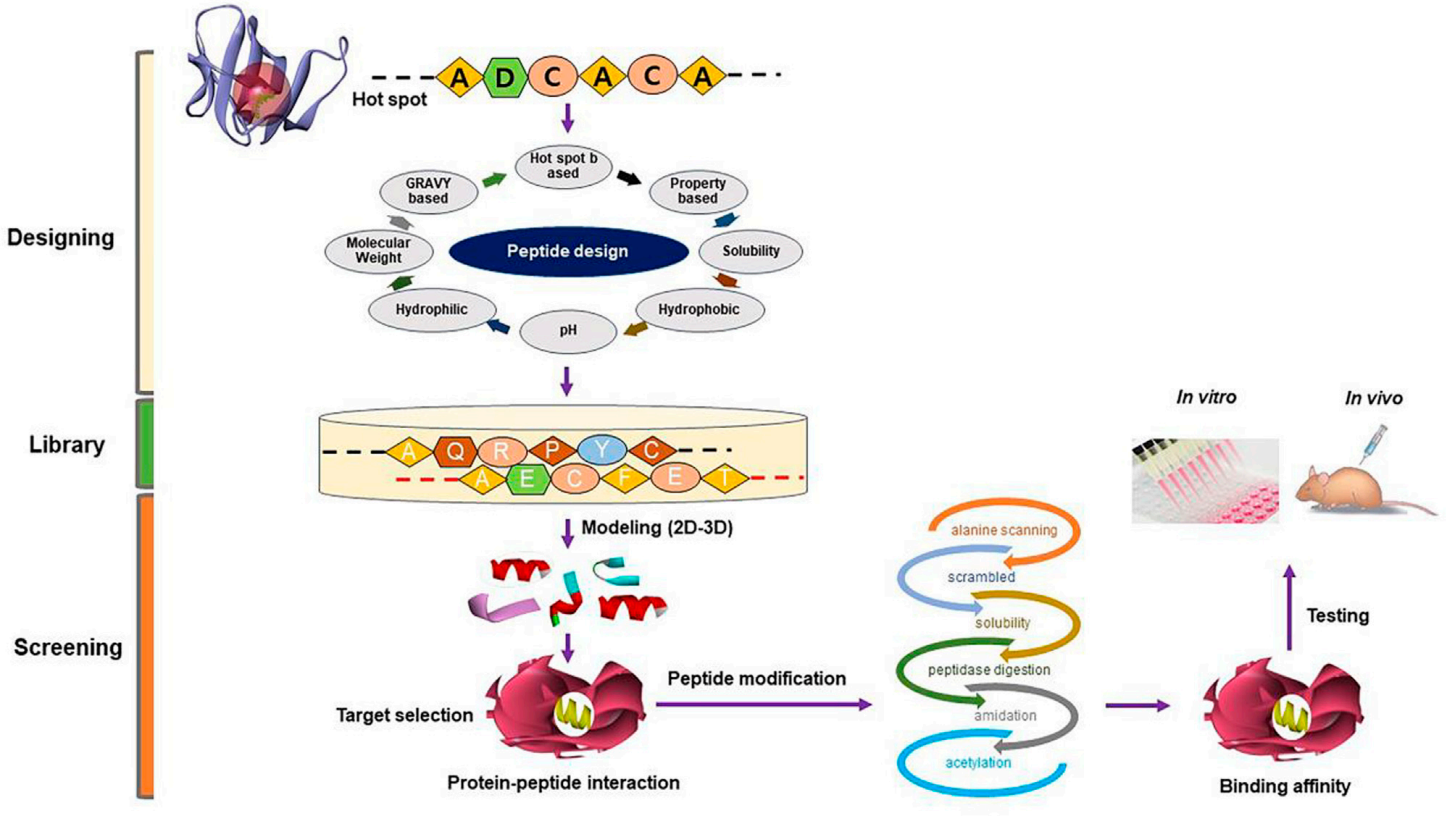
A flowchart depicting the workflow for in silico peptide design.

We have been working in the SM field using *in silico*, *in vitro*, and *in vivo* techniques with an emphasis on the mechanism responsible for SM development and regeneration, for more than a decade. Our quest for an efficacious natural MSTN inhibitor in the form of a small molecule or short peptide is ongoing. This section will give a brief understanding of screening natural compounds (Figure 2) and designing short peptides (Figure 3).

### Protein-Protein Interactions

PPIs play a vital role in mediating various cellular processes, and thus, have attracted research attention (Guo et al., 2014; Qiu et al., 2020). The advancement in the field of 3-dimensional structure predictions of proteins and PPI includes the recent discovery of AlphaFold (Jumper et al., 2021) and AlphaFold2 (Bryant et al., 2022). Studies have shown that PPI provides a means of effectively regulating various pathways and of developing therapeutic targets. However, all the interfaces of proteins do not contribute equally to PPI (Guo et al., 2014).

The mechanism of action of MSTN is based on its interaction with other proteins in the pathway leading to the transformation of latent MSTN to its mature form and further activation of the Smad pathway, which leads to the inhibition of myogenesis. Studies have shown PPIs are key mediators of various signaling and regulatory networks (Villoutreix et al., 2008). As discussed above, during the activations of different signaling processes, and thus, the activations of atrophic genes, MSTN interacts with a large number of different proteins, which provides clues for the design of peptide inhibitors of MSTN. Peptides that inhibit MSTN activity can be derived in two ways.

### Derivation of Peptide Inhibitors From MIPs

MSTN is known to interact with FST, GASP-1, GASP-2, decorin, FMOD, and FLRG, which are collectively referred to as MIPs. These proteins inhibit the formation of mature MSTN complex and interfere with complex formation between MSTN and ACVRIIB**.** Designing short peptides based on the make-up of MIPs offers a potential means of effective MSTN inhibitors, and some studies indicate that these inhibitory peptides have therapeutic potential for the treatment of a range of muscular dystrophies (Tsuchida, 2008). FS I-I (MSTN-specific inhibitor derived from FST) provides an example of an FST-derived inhibitory peptide and increased SM mass in mdx/FS I-I mice and reduced cell infiltration into muscles (Tsuchida, 2008). DCN is another MIP protein with MSTN inhibitory potential. DCN48-71 and 42-65 are two short fragment peptides derived from members of the small leucine-rich proteoglycan family that demonstrated MSTN inhibitory activity *in vitro* (El Shafey et al., 2016). Similarly, other studies have successfully identified and tested short peptides capable of inhibiting MSTN activity. One such example is WRQNTRYSRIEAIKIQILSKLRL-amide, which was designed based on the mouse MSTN prodomain. Administration of this peptide to MDX mice (a model of DMD) significantly increased muscle mass. Subsequently, several peptides were designed by structure-activity relationship (SAR) analysis of this short peptide, and peptide ‘3d' (XRQNTRYSRIEWIKIQIISKLRL-amide) exhibited 11 times the potency of its parent and induced muscle growth in MDX and wild-type ICR mice (Takayama et al., 2017).

Detailed structural study of interactions between MIPs and MSTN helped identify residues largely responsible for inhibiting MSTN activity. These were later assembled in different combinations to produce candidate anti-MSTN peptides. Three-dimensional (3D) structures of MSTN in complex with various isomers of FST are available in the protein data bank (PDB) (e.g. PDB ID: 3HH2, 3SEK, and 5JHW) (Cash et al., 2012). Crystal structure analysis showed that the binding of two Fst-like 3 (FSTL3) molecules with MSTN dimer resulted in a highly compact structure and strong interactions in the docked complex.

### Self-Inhibitory Peptides

Designing peptide inhibitors targeting PPIs is challenging due to the large sizes of PPIs. Nonetheless, substantial progress has been reported in the field of PPI inhibitor design during the last few years (Jones and Thornton, 1996; Lu et al., 2020; Valtonen et al., 2020). The use of self-derived peptide inhibitors has been one of the most successful PPI inhibitor design strategies. This strategy involves deriving inhibitory peptides from PPIs that act by inhibiting their cognate interactions. The use of self-inhibitory peptides has attracted much interest as a means of inhibiting PPIs that are considered important therapeutic targets (Vlieghe et al., 2010). MSTN activity can also be inhibited by disrupting the interaction between MSTN and its receptor. The approach of targeting protein-protein interfaces to block interactions between MSTN-ACVRIIB instead of enzyme active sites provides another way of reducing MSTN-mediated signaling activity without hampering the intrinsic catalytic functionality of these proteins (Chen et al., 2021).

### Virtual Screening for Natural Antagonists of MSTN

Computer-aided drug design (CADD) and computer-assisted molecular design (CAMD) are used as drug discovery tools in the pharmaceutical science field (Baig et al., 2016). VS. is widely used for drug discovery and is complemented by High Throughput Screening (HTS). The VS./HTS approach is used to screen compound libraries quickly and cost-effectively using high-end computational approaches. Selected compounds are subsequently tested for biological activity. The activities of many identified natural compounds have yet to be determined, such as those detailed in the Chinese traditional medicine and Korean medicinal compound databases. Several groups have attempted to identify novel therapeutic candidates that target MSTN, but unfortunately, a large number of identified compounds were not MSTN specific and were also found to block activin A and TGF-β signaling (Suh and Lee, 2020a). The specific targeting of MSTN remains a significant research challenge as many TGF-β ligands exhibit considerable structural similarities. Recently, we performed a VS-based analysis on known muscle-enhancing natural compounds for MSTN inhibitory activity and identified curcumin and gingerol as candidates (Baig et al., 2017). Undoubtedly, there are limitations of the VS approach that should be taken into account. The prevalence of stereochemical and valence mistakes in biochemical compound libraries may potentially result in inviable molecules (Williams et al., 2012; Santana et al., 2021). There are a variety of open source and licensed virtual screening software/tools available, and each has its own constraints that must be overcome to prevent the production of erroneous conclusions or artifacts (Gimeno et al., 2019). For VS, licensed software such as Molecular Operating Environment (MOE) (Vilar et al., 2008), and the GLIDE module in Schrodinger (Bhachoo and Beuming, 2017) as well as open access tools such as Autodock Vina (Trott and Olson, 2010), are commonly used.

## Conclusion

The inhibitory role played by MSTN in muscle development has made it an important therapeutic target for accelerating muscle mass. There are several ways of disrupting MSTN activity ranging from the use of MSTN antibodies to natural compounds. Detailed knowledge of these strategies and the use of *in silico* techniques should improve knowledge of the structural characteristics of MSTN and its bindings with inhibitory proteins, derived inhibitors, and other natural compounds. Structural insight of binding between different MIPs and MSTN should open new doors to the design of better therapeutic peptide candidates. Although no drugs have yet been developed to prevent muscle degeneration, we believe that research efforts targeting MSTN will result in treatments that attenuate muscle degeneration and improve the quality of life in the elderly and those suffering from MD.
